# Evaluating periapical and periodontal health in root canal treated teeth using various obturation methods

**DOI:** 10.6026/973206300213175

**Published:** 2025-09-30

**Authors:** Priyanshi Kankriya, Gaurav Garg, Anupama Ahirwar, Anjali Gupta, Subasish Behera, Soumyaranjan Nanda, Miral Mehta, Jugajyoti Pathi

**Affiliations:** 1 Department of Conservative Dentistry and Endodontics, Pacific Dental College and Hospital, PAHER University, Debari, Udaipur, Rajasthan, India; 2 Department of Conservative Dentistry and Endodontics, Bankey Bihari Dental College, Ghaziabad, Uttar Pradesh, India; 3 Department Of Dentistry, Sunderlal Patwa Government Medical College, Mandsaur, Madhya Pradesh, India; 4 Department of Conservative & Endodontics, Maitri College of Dentistry and Research Centre, Anjora, Durg, Chhattisgarh, India; 5 Department of Conservative Dentistry and Endodontics, Government Dental College and Hospital (SCB), Manglabag, Cuttack, Odisha, India; 6 Endodontics, Private Practitioner, Cuttack, Odisha, India; 7 Department of Pediatric and Preventive Dentistry, Karnavati School of Dentistry, Karnavati University, Gandhinagar, Gujarat, India; 8 Department of Oral & Maxillofacial Surgery, Kalinga Institute of Dental Sciences, KIIT Deemed to be University. Bhubaneswar, Odisha, India

**Keywords:** Root canal treatment, obturation techniques, warm vertical compaction, periapical healing, periodontal health

## Abstract

Effective root canal obturation is critical for preventing reinfection and ensuring long-term periapical and periodontal health. This randomized controlled trial evaluated 120 mandibular molars treated with cold lateral compaction (CLC), warm vertical compaction (WVC), or single-cone with bioceramic sealer (SBC). At 12 months, WVC showed significantly greater PAI score reduction (2.85 ± 0.51) and higher complete healing rates (87.5%) compared to CLC (65.0%) and SBC (72.5%) (p=0.018). Periodontal parameters (PD, CAL, BOP, PI) improved significantly across all groups with no inter-group differences. Thus, we show WVC offers superior periapical healing while maintaining periodontal stability.

## Background:

Root canal treatment (RCT) is fundamental to endodontic therapy, designed to conserve teeth affected by pulpal and periapical pathosis [1]. The principal aims of root canal therapy (RCT) are comprehensive chemo mechanical debridement of the root canal system, efficient disinfection and three-dimensional obturation to avert reinfection and promote periapical healing [2]. Obturation, the last stage of root canal therapy, entails filling the cleansed and shaped root canal with biocompatible materials to establish a hermetic closure, therefore entombing leftover germs and preventing microleakage at the coronal or apical ends [3]. The quality of obturation is considered a crucial factor in long-term therapeutic success [4]. A variety of obturation techniques have been created and refined throughout the years, each possessing unique advantages and limitations. Cold lateral compaction (CLC) has always been regarded as the gold standard owing to its reliability, relative ease of execution and cost efficiency [5]. Nonetheless, apprehensions persist about its capacity to thoroughly occupy intricate anatomical variations, including isthmuses, auxiliary canals and apical deltas, which may result in voids that could host bacteria [6]. Warm vertical compaction (WVC), employing heated gutta-percha, seeks to attain a more uniform, three-dimensional fill by conforming the material closely to canal imperfections by thermoplastic flow [7]. Advocates contend that this greater adaptability results in superior sealing capability and better long-term effects [8]. The single-cone method (SCT), especially when combined with contemporary bioceramic sealers, has lately acquired prominence for its efficacy and simplicity [9]. Bioceramic sealers demonstrate superior biocompatibility, antibacterial characteristics and the capacity to generate hydroxyapatite, which may enhance bioactivity and sealing efficacy [10]. The dependence on a singular master cone prompts inquiries on its efficacy in attaining equivalent canal space filling and sealing compared to methods employing many cones or compaction pressures, particularly in non-round canals [11].

Recent research has shown contradictory findings concerning the relative effectiveness of different approaches. Although certain *in vitro* and *ex vivo* investigations indicate that WVC offers enhanced apical sealing and adaptation relative to CLC and SCT [12, 13], other studies demonstrate similar sealing efficacy, especially when bioceramic sealers are employed with SCT [14, 15]. Clinical research directly comparing long-term periapical healing outcomes among these three procedures is relatively limited. The possible influence of the obturation technique on periodontal health metrics, including probing depth and clinical attachment level, has been inadequately explored. The major emphasis of obturation is intracanal; however, the technique's impact on the quality of the coronal seal and the potential extrusion of materials may theoretically affect the periodontal attachment mechanism [16]. A notable research deficiency is present due to the absence of comprehensive, long-term clinical trials that directly compare the periapical healing efficacy and periodontal health outcomes of CLC, WVC and SCT utilizing bioceramic sealer. Most existing comparative studies are conducted in laboratory settings or concentrate on short-term radiography outcomes without thorough periodontal evaluation [17]. Therefore, it is of interest to assess and compare the periapical healing response and periodontal health outcomes in teeth receiving primary root canal treatment via cold lateral compaction, warm vertical compaction and the single-cone technique with bioceramic sealer over 12-month duration.

## Materials and Methods:

## Study design and setting:

This prospective, randomized, controlled clinical trial was conducted at the Department of Conservative Dentistry and Endodontics between January 2021 and December 2022.

## Sample size calculation:

Based on a pilot study and previous literature, the expected difference in complete periapical healing rates (PAI ≤2) between the best (WVC ~85%) and worst (CLC ~65%) performing groups was 20%. Assuming a power of 80% (β=0.2), an alpha level of 0.05 and accounting for a potential 15% dropout rate, a minimum sample size of 36 patients per group was calculated. To enhance robustness, 40 patients were enrolled per group, totalling 120 participants.

## Participant selection:

## Inclusion criteria:

Systemically healthy adults (18-65 years); presence of a non-vital permanent mandibular first or second molar requiring primary RCT due to irreversible pulpitis or pulp necrosis with radiographic evidence of periapical radiolucency (PAI score 3 or 4); tooth restorable with a full-coverage crown post-RCT; absence of significant periodontal disease (probing depth ≤4mm, no furcation involvement >Grade I, no radiographic bone loss >1/3 root length); patient willing and able to comply with follow-up schedule.

## Exclusion criteria:

Pregnancy or lactation; severe systemic disease (uncontrolled diabetes, immunocompromised status); history of antibiotic use within 3 months prior to RCT; tooth with internal/external root resorption, immature apex, or perforation; presence of a sinus tract; inability to achieve adequate rubber dam isolation; tooth requiring post-retained core; patient allergic to gutta-percha, zinc oxide-eugenol, or bioceramic materials.

## Randomization and blinding:

Eligible participants were randomly assigned to one of three obturation groups using a computer-generated block randomization sequence (block size of 6) concealed in opaque, sequentially numbered envelopes. The envelope was opened immediately after canal preparation was completed and deemed adequate. The outcome assessor (radiographic and clinical examiner) and the statistician were blinded to the group allocation. The treating endodontist performing the RCT could not be blinded due to the nature of the techniques.

## Clinical procedures:

All RCTs were performed by a single experienced endodontist (5+ years) under standardized protocols:

[1] Anesthesia and Isolation: Local anesthesia (2% lidocaine with 1:100,000 epinephrine) was administered. Teeth were isolated using a rubber dam.

[2] Access Cavity: Standard endodontic access cavities were prepared using high-speed diamond burs under water cooling.

[3] Canal Negotiation and Working Length: Canals were negotiated with size 10 K-files. Working length (WL) was determined electronically using a root canal apex locator (Root ZX II, J. Morita Corp.) and confirmed radiographically.

[4] Chemo mechanical Preparation: Canals were prepared using a rotary nickel-titanium system (ProTaper Gold, Dentsply Sirona) to a standardized master apical file size (size 25 for mesial canals, size 30 for distal canals, 0.06 taper). Irrigation was performed with 5.25% NaOCl (30ml per canal) using a side-vented needle, activated with passive ultrasonic irrigation (PUI, 3x20s per canal). Final rinse included 17% EDTA (1ml, 1min) followed by a final flush with sterile saline. Canals were dried with paper points.

[5] Obturation:

[6] Group 1 (CLC): Canals were coated with AH Plus sealer (Dentsply Sirona). A standardized master gutta-percha cone (size 25/0.06 or 30/0.06) was fitted to WL. Accessory fine-medium gutta-percha cones were laterally compacted using a finger spreader until the canal was densely filled. Excess gutta-percha was seared off and condensed vertically.

[7] Group 2 (WVC): Canals were coated with AH Plus sealer. A standardized master gutta-percha cone was fitted to WL. A System B Heat Source (SybronEndo) was used to vertically compact the apical 3-4mm of the master cone. The remaining canal was backfilled using the Obtura II system (Obtura Spartan) with injectable thermoplasticized gutta-percha (180°C), followed by vertical compaction with pluggers.

[8] Group 3 (SBC): Canals were coated with a bioceramic sealer (EndoSequence BC Sealer, Brasseler USA). A single gutta-percha cone matching the final rotary instrument size and taper (size 25/0.06 or 30/0.06) was seated to WL. No compaction was performed.

[9] Coronal Seal: All teeth received a temporary restoration (Cavit G, 3M ESPE) immediately after obturation. Patients were scheduled for permanent full-coverage crown placement within 4 weeks.

## Outcome assessment:

[1] Periapical Healing: Standardized periapical radiographs (paralleling technique using Rinn holders) were taken at baseline (pre-op), 6 months and 12 months post-obturation. All radiographs were digitized and evaluated by two independent, calibrated examiners blinded to the group allocation. Periapical status was assessed using the Periapical Index (PAI) score (1-5). Inter-examiner reliability was assessed using Cohen's Kappa (κ=0.88). Disagreements were resolved by consensus. Healing was defined as a reduction in PAI score. Complete healing was defined as a PAI score of 1 or 2.

[2] Periodontal Health: Clinical periodontal examination was performed at baseline and 12 months by a single calibrated periodontist blinded to the group allocation. Parameters recorded at six sites per tooth (mesiobuccal, midbuccal, distobuccal, mesiolingual, midlingual, distolingual) included:

[3] Probing Depth (PD): Measured in millimeters (mm) using a University of North Carolina (UNC) 15 periodontal probe.

[4] Clinical Attachment Level (CAL): Calculated as the distance from the cementoenamel junction (CEJ) to the base of the sulcus/pocket (mm).

[5] Bleeding on Probing (BOP): Recorded as present (1) or absent (0) within 30 seconds after probing. The percentage of sites bleeding per tooth was calculated.

[6] Plaque Index (PI): Assessed using the Silness and Löe index (0-3) at four sites per tooth; mean score per tooth was calculated.

## Statistical analysis:

Data were analyzed using SPSS Statistics version 27.0 (IBM Corp.). Normality of distribution was assessed using the Shapiro-Wilk test. Descriptive statistics (mean ± standard deviation, frequencies, percentages) were calculated for all variables. Intergroup comparisons at baseline were performed using ANOVA for continuous variables (age, PD, CAL, PI) and Chi-square test for categorical variables (gender, tooth type, PAI distribution). For PAI scores over time, repeated measures ANOVA was used with Bonferroni post-hoc tests. Chi-square test compared the proportion of teeth achieving complete healing (PAI ≤2) at 12 months between groups. Intergroup comparisons of periodontal parameters at 12 months were performed using ANOVA (PD, CAL, PI) and the Kruskal-Wallis test (BOP%). Paired t-tests analyzed changes in periodontal parameters within each group from baseline to 12 months. A p-value < 0.05 was considered statistically significant.

## Results:

A total of 156 patients were screened; 120 met the inclusion criteria and were randomized (40 per group). All participants completed the 12-month follow-up. Baseline demographic and clinical characteristics were comparable across the three groups (Table 1 - see PDF). There were no significant differences in age (p=0.621), gender distribution (p=0.912), tooth type (p=0.875), baseline PAI scores (p=0.734), or baseline periodontal parameters (PD: p=0.538; CAL: p=0.612; BOP%: p=0.489; PI: p=0.571). All groups showed a significant reduction in mean PAI scores from baseline to 6 months and 12 months (p<0.001 for all groups). However, the rate and extent of healing differed significantly between groups. Repeated measures ANOVA revealed a significant group effect (p<0.001), time effect (p<0.001) and group-time interaction (p<0.001). At 6 months, the mean PAI score reduction was significantly greater in the WVC group (1.95 ± 0.48) compared to CLC (1.30 ± 0.62) and SBC (1.55 ± 0.59) (p<0.001). At 12 months, the mean PAI score reduction was significantly greater in the WVC group (2.85 ± 0.51) compared to CLC (2.10 ± 0.72) and SBC (2.35 ± 0.68) (p<0.001). The difference between CLC and SBC at 12 months was not statistically significant (p=0.082). The proportion of teeth achieving complete healing (PAI ≤2) at 12 months was significantly higher in the WVC group (35/40, 87.5%) compared to the CLC group (26/40, 65.0%) and the SBC group (29/40, 72.5%) (p=0.018). The difference between CLC and SBC was not significant (p=0.465). At 12 months, there were no statistically significant differences between the three groups for any periodontal parameter (Table 2 - see PDF). Mean PD, CAL, BOP% and PI values were comparable across CLC, WVC and SBC groups (PD: p=0.412; CAL: p=0.385; BOP%: p=0.527; PI: p=0.491). Within each group, significant improvements in PD and CAL were observed from baseline to 12 months (p<0.001 for all groups). Mean PD decreased by 0.25-0.30mm and mean CAL decreased by 0.20-0.25mm across groups. BOP% and PI showed slight numerical decreases, but these were not statistically significant within groups (p>0.05 for all).

## Discussion:

The enhanced periapical healing noted with WVC corresponds with its core premise of attaining a compact, three-dimensional fill via the flow of thermoplasticized gutta-percha. This method is especially proficient in addressing intricate anatomical structures such as isthmuses, lateral canals and apical ramifications, frequently observed in mandibular molars [18]. The improved adaptability and less void formation likely reinforce the barrier against microleakage, hence reducing the chance of residual or re-entering bacteria that could sustain periapical inflammation [19]. Our results validate numerous clinical studies indicating superior success rates for WVC in comparison to CLC, particularly in teeth exhibiting intricate anatomy or pre-operative periapical abnormalities [20, 21]. A recent comparative study by Kahate *et al.* likewise demonstrated significant improvements in periapical healing and maintenance of periodontal health with different obturation techniques, further supporting the clinical reliability of warm vertical compaction [22]. The markedly enhanced healing noted at 6 months in the WVC group indicates a more prompt and vigorous biological response, possibly associated with the improved initial seal. The SBC approach demonstrated intermediate performance in this investigation, exhibiting considerably superior healing compared to CLC at 6 months, but not at 12 months and significantly inferior healing compared to WVC at both time intervals. Although bioceramic sealers possess beneficial characteristics such as biocompatibility, bioactivity and antimicrobial properties [23], dependence on a singular master cone without compaction may restrict their efficacy in achieving comparable canal space filling to WVC, especially in oval or irregular canals frequently found in the mesial roots of mandibular molars [24]. Our findings diverge from several laboratory studies indicating similar sealing efficacy between SBC with bioceramic sealer and WVC [25]. This contrast underscores the possible constraints of *in vitro* studies in forecasting intricate *in vivo* biological responses that encompass host defense mechanisms, bacterial interactions and temporal tissue regeneration. The reported healing rate of 72.5% for SBC is clinically acceptable; nonetheless, it indicates that although bioceramic sealers are advantageous, they may not entirely mitigate the potential inadequacy of space-filling associated with the single-cone technique in complex cases. Additional long-term research is necessary.

The CLC group exhibited the worst periapical healing results. Although a dependable method, CLC is recognized as being less efficacious than WVC in addressing canal abnormalities and may result in a greater number of voids, especially in the apical third and regions of canal complexity [26]. These holes may serve as habitats for bacterial survival and proliferation, potentially obstructing complete periapical resolution [27]. The observed healing rate of 65%, albeit lower than that of other groups, is within the range documented for CLC in certain clinical investigations, particularly with teeth with pre-operative periapical lesions [28]. Our findings strongly indicate that WVC is preferable to CLC for optimizing periapical healing in molars. This study demonstrated no significant variations in periodontal health markers (PD, CAL, BOP, PI) across the three obturation procedures after 12 months. All groups exhibited modest yet substantial enhancements in probing depth (PD) and clinical attachment level (CAL) from baseline, possibly due to the eradication of the intracanal infection source and the implementation of a properly sealed permanent repair, thereby diminishing inflammatory stimuli [29]. The absence of inter-group differences indicates that the obturation technique, when executed properly and accompanied by a final coronal seal, does not negatively impact the periodontal attachment mechanism. This discovery is comforting, as apprehensions have been expressed over the possible ejection of sealer or gutta-percha during obturation (especially with WVC), aggravating periodontal tissues [30]. The precise method utilized, encompassing regulated sealer application and compaction pressures, probably reduced such threats. The same plaque levels among groups suggest that patient oral hygiene and the quality of the final restoration were constant factors affecting periodontal health, outweighing any modest variances associated with the obturation process.

## Conclusion:

Warm vertical compaction (WVC) showed significantly superior periapical healing compared to CLC and SBC, while all techniques maintained similar periodontal health within the 12-month study period. WVC is recommended as the preferred obturation method, especially in complex anatomies. Long-term studies are needed to validate the durability of these outcomes.
